# Metal Hypersensitivity in Total Joint Arthroplasty: Molecular Immunopathogenesis, Diagnostic Controversies, and Prospects for Precision Medicine

**DOI:** 10.3390/ijms27198570

**Published:** 2026-09-25

**Authors:** Nikodem Kuczyński, Bartłomiej Szewczyk, Michał Niedźwiedź, Marcin Kurowski, Katarzyna Poznańska-Kurowska

**Affiliations:** 1 Faculty of Medicine, Collegium Medicum, The Mazovian University in Płock, 09-402 Płock, Poland; n.kuczynski@mazowiecka.edu.pl; 2 Department of Anatomical Dissection and Plastination, The Mazovian University in Płock, 09-402 Płock, Poland; 3 Department of Dermatology, Pediatric and Oncological Dermatology, Dr W Bieganski Regional Hospital, 91-347 Łódź, Poland; mniedzwiedz@bieganski.com.pl (M.N.); katarzyna.poznanska@imp.lodz.pl (K.P.-K.); 4 Department of Immunology and Allergy, Medical University of Łódź, 92-213 Łódź, Poland; 5 Nofer Institute of Occupational Medicine, Clinic of Occupational Diseases and Environmental Health, 91-348 Łódź, Poland

**Keywords:** metal hypersensitivity, total joint arthroplasty, molecular immunology, human leukocyte antigen (HLA), delayed-type hypersensitivity, implant failure, patch testing

## Abstract

Implant-related metal hypersensitivity (MH) after total joint arthroplasty (TJA) is an uncommon and diagnostically challenging complication in which antigen-specific T-cell responses may coexist with nonspecific inflammatory reactions to wear and corrosion products. This narrative review critically summarizes current evidence on the molecular immunopathogenesis, clinical presentation, diagnosis, and management of suspected MH, with particular emphasis on HLA-associated susceptibility and the 2023 European Academy of Allergy and Clinical Immunology (EAACI) mechanistic nomenclature. A structured search of PubMed, Scopus, and Web of Science was used to identify relevant clinical, immunological, and consensus literature. Metal sensitization is substantially more common than clinically relevant implant-associated hypersensitivity, and positive patch or lymphocyte transformation tests do not by themselves establish causality. Current evidence supports selective, clinically contextualized testing after exclusion of infection and mechanical causes; routine preoperative screening in patients without a suggestive history is not recommended. HLA class II associations and metal–peptide–MHC/T-cell receptor interactions provide a plausible molecular basis for interindividual susceptibility, but available genetic data remain insufficient for clinical risk stratification or implant selection. Future studies should integrate standardized phenotyping, quantitative metal exposure, histopathology, and molecular immune profiling before implementing precision arthroplasty.

## 1. Introduction

Total joint arthroplasty (TJA) has become a routine, highly effective procedure for treating end-stage degenerative joint disease [1,2]. More than one million procedures are performed annually worldwide [2]. Human leukocyte-based studies have demonstrated metal reactivity in patients with hip implants [3], while projections indicate a substantial further increase in arthroplasty demand [4]. TJA provides durable pain relief, functional improvement, and enhanced quality of life [5]; however, revision rates remain significant despite advances in surgical technique and implant design [5,6]. The growing number of primary and revision arthroplasties is expected to markedly increase the population exposed to orthopedic implant materials and, consequently, the clinical relevance of implant-related complications, including the rare and debated entity of metal hypersensitivity (MH) [6,7].

Modern orthopedic implants predominantly consist of metal alloys with favorable biomechanical strength and biocompatibility. Nevertheless, implanted metals undergo corrosion and wear, leading to the release of degradation products, particularly metal ions [8,9,10]. These ions may function as haptens by binding endogenous proteins and forming metal–protein complexes capable of eliciting delayed-type (type IV) hypersensitivity reactions mediated by T lymphocytes [3,11,12]. Nickel represents the most common sensitizing metal in humans [13], followed by cobalt and chromium, while titanium is generally considered less immunogenic [3,10]. Recent large-database evidence associated a preoperative diagnosis of MH with inferior implant survivorship after TKA, but not THA; however, this observational association does not establish causality [14]. Following exposure to metal antigens, peripheral T lymphocytes, particularly Th1 cells, become activated and release pro-inflammatory cytokines including tumor necrosis factor-α (TNF-α), interferon-γ (IFN-γ), interleukin-1 (IL-1), and interleukin-2 (IL-2), resulting in macrophage recruitment and the establishment of a pro-inflammatory microenvironment that may impair implant biocompatibility and osseointegration [15,16].

Beyond local tissue responses, metal degradation products and activated immune cells may disseminate systemically, potentially contributing to remote clinical manifestations [17,18]. In a 26-year Turkish cohort of 2932 patients referred for patch testing, sensitization to baseline metals was detected in 28.4% of patients, most frequently to nickel (21.1%); these referral-based estimates should not be interpreted as population prevalence [19]. Randomized comparisons of coated and uncoated TKA components have not demonstrated consistent clinically relevant outcome differences attributable to metal sensitization [20]. Previous reviews nevertheless indicate that cutaneous metal sensitization is common and may be detected more frequently in selected implant populations [21,22]. Clinically coded MH remains uncommon: a national database study reported diagnoses in 0.133–0.165% of TKA patients, and patients with an MH diagnosis accounted for only 0.68% of all TKA revisions [23]. Evidence concerning clinical outcomes remains heterogeneous; a meta-analysis of broadly defined allergies found modestly worse patient-reported outcomes, whereas arthroplasty-specific reviews have not established a consistent causal relationship [24,25,26]. Poor concordance among available testing modalities further contributes to uncertainty regarding whether sensitization precedes implant failure or develops secondarily [27].

The historical orthopedic literature often summarizes a gradient in metal sensitization prevalence of approximately 10–15% in the general population, about 20–25% in patients with well-functioning implants, and roughly 50–60% in selected cohorts with poorly functioning or failed implants [15,17,28]. These figures should be interpreted cautiously because they derive from heterogeneous studies using different tests, thresholds, implant types, and often symptom- or failure-enriched populations. They therefore do not represent the incidence of clinically causal implant hypersensitivity and cannot resolve directionality: sensitization may precede failure in some patients, whereas increased wear, corrosion, and metal exposure from a failing implant may promote secondary sensitization in others.

Emerging evidence suggests that host genetic factors, particularly variations within the human leukocyte antigen (HLA) system involved in antigen presentation, may influence individual susceptibility to metal-induced immune responses and could partially explain the variability observed among patients exposed to similar implant materials [29,30,31].

The increased frequency of metal sensitization reported in some arthroplasty cohorts has raised questions about its relationship to implant failure and highlights the need for accurate diagnostic evaluation to guide material selection in primary and revision procedures [7,32]. Recent evidence remains inconsistent: matched database analyses have identified an association between a preoperative MH diagnosis and revision after TKA but not THA, whereas meta-analytic findings concerning broadly defined allergies show only modest differences in patient-reported outcomes [6,14,24]. These observations do not establish that hypersensitivity is a direct cause of implant failure. Consequently, MH remains one of the most controversial topics in joint arthroplasty.

Diagnostic assessment of implant-related MH presents significant challenges [17]. Patch testing has historically been the most widely used clinical test for contact sensitization [21]; however, it relies on cutaneous antigen presentation by Langerhans cells, which may not adequately reflect the immunologic environment of the periprosthetic space [3]. Existing assessment frameworks distinguish cutaneous testing from laboratory methods intended to evaluate systemic cell-mediated responses [33]. Moreover, a recent cohort of 225 patients referred for suspected prosthesis-related hypersensitivity showed that clinical presentation and comorbidities alone were insufficient to identify patients with positive patch-test findings [34]. Peripheral mononuclear cells, including lymphocytes and monocytes, are more likely to function as antigen-presenting cells around implants, prompting the development of in vitro functional assays such as lymphocyte proliferation tests, cytokine production assays, migration inhibition tests, lymphocyte transformation tests (LTT), and the Memory Lymphocyte Immunostimulation Assay (MELISA) [35,36].

Given the projected rise in arthroplasty procedures and revisions, understanding the immunopathogenesis, clinical significance, and optimal diagnostic strategies for MH is increasingly important. Better identification of at-risk patients could support personalized implant selection, reduce revision burden, and improve clinical outcomes and healthcare cost-effectiveness.

## 2. Literature Search Strategy

This study was conducted as a narrative review of the current literature on MH in TJA, with a particular focus on immunopathogenesis, HLA susceptibility, diagnostic approaches, and clinical management. A structured literature search was performed using major medical databases, including PubMed, Scopus, and Web of Science, to identify relevant studies published up to July 2026. Search terms included combinations of “metal hypersensitivity,” “total joint arthroplasty,” “HLA susceptibility,” “patch test,” “lymphocyte transformation test,” “implant failure,” and “EAACI classification.”

Eligible sources included clinical studies, systematic reviews, meta-analyses, expert consensus statements, and mechanistic human or in vitro studies relevant to the scope of this article. English-language publications were prioritized, while non-English sources were considered when they contributed directly to the immunological or clinical questions addressed. Animal studies and conference abstracts without available full texts were excluded.

Articles were screened by title and abstract and then assessed in full text for relevance to the review questions. When evidence overlapped or conflicted, greater weight was given to more recent studies, larger or better-characterized cohorts, methodologically stronger evidence, and peer-reviewed consensus guidance. Reference lists of included articles and recent reviews were hand-searched for additional relevant publications. Two authors independently screened titles/abstracts and reviewed full texts, with disagreements resolved by consensus.

We synthesized findings qualitatively, emphasizing consistent observations, emerging immunogenetic trends (such as HLA profiling), and ongoing controversy in the diagnosis and management of implant-related MH. Because this review is narrative, no formal meta-analysis or quantitative synthesis was performed.

## 3. Modern Nomenclature of Hypersensitivity (EAACI 2023)

The recent EAACI position paper proposes an updated nomenclature and classification of hypersensitivity reactions that expands the traditional Gell and Coombs I–IV system [37] to better reflect current immunological knowledge and clinical practice. The revised framework expands the classical scheme into an updated set of mechanistic categories, including three distinct type IV cellular response patterns.

Type I hypersensitivity remains defined as IgE-mediated immediate reactions involving mast cell and basophil activation. Type II hypersensitivity refers to IgG or IgM antibody-mediated cytotoxic or receptor-modulating reactions directed against cell-surface or extracellular matrix antigens. Type III hypersensitivity involves immune complex-mediated inflammation with complement activation and tissue deposition. Type IV hypersensitivity is defined as a delayed T cell-mediated immune response with three dominant functional immune patterns (Table 1):

### 3.1. Type Iva–T1 Immune Response

This pathway is mediated predominantly by memory Th1 and Tc1 cells. IL-12 and IFN-γ are central to T1 polarization and activation, whereas IL-23 may support maintenance or effector responses but does not directly drive primary differentiation of naive T cells into Th1/Tc1 lineages [38]. Effector mechanisms include IFN-γ, TNF-α and lymphotoxin production, macrophage activation and granuloma formation [39]. Classically activated macrophages release reactive oxygen species, proteases and pro-inflammatory cytokines that contribute to tissue injury [40]. Tc1 cells additionally mediate cytotoxicity through perforin, granzyme B and death-receptor signalling [41]. Typical clinical manifestations include allergic contact dermatitis, chronic hypersensitivity pneumonitis and celiac disease [40].

### 3.2. Type IVb–T2 Immune Response

This pathway is driven by Th2 cells, ILC2 and eosinophils, with key cytokines IL-4, IL-5 and IL-13. IL-4 and IL-13 promote IgE class switching [42], whereas IL-5 mediates eosinophil maturation, recruitment and survival [43]. Activated eosinophils release cytotoxic granule proteins and form extracellular traps, leading to epithelial injury and chronic inflammation [43]. Type IVb responses are characteristic of chronic airway and skin allergic diseases, including asthma, allergic rhinitis, chronic rhinosinusitis and atopic dermatitis [40].

### 3.3. Type IVc–T3 Immune Response

This subtype is dominated by Th17, Tc17, and ILC3 cells that produce IL-17 family cytokines [40]. IL-17-mediated activation of stromal and innate immune cells induces IL-8 production and neutrophil recruitment [44], which represents the hallmark of type IVc hypersensitivity. Neutrophil-derived enzymes, reactive oxygen species and neutrophil extracellular traps contribute to tissue damage [45]. Type IVc responses frequently coexist with IVa inflammation but may dominate in selected neutrophilic inflammatory diseases [46].

Importantly, the EAACI 2023 framework extends hypersensitivity into nine mechanistic categories: types I–III are antibody-mediated; types IVa, IVb, and IVc represent T1-, T2-, and T3-dominant cell-mediated responses; type V is associated with epithelial barrier defects; type VI with metabolic-induced immune dysregulation; and type VII with direct cellular and inflammatory responses to chemical substances. Mixed and overlapping mechanisms may occur in clinical disease [40].

This updated nomenclature is useful as a mechanistic framework, but it should not be interpreted as evidence that all of these pathways have been demonstrated in implant-related metal hypersensitivity. In this context, its main value is to distinguish established type IV T-cell biology from broader tissue-driven or chemical inflammatory mechanisms that may coexist around an implant.

## 4. Materials Used in Total Hip and Knee Arthroplasty

Common metallic biomaterials used in orthopedics include 316L stainless steel, cobalt–chromium–molybdenum alloys, in which cobalt constitutes the balance with approximately 27–30% chromium and 5–7% molybdenum, and titanium alloys such as Ti-6Al-4V. Exact elemental composition varies by applicable alloy standard and manufacturer [10]. Rare hypersensitivity-related failure has also been described after use of a cobalt-containing great-toe hemi-implant [47]; however, a single case outside large-joint arthroplasty cannot establish the frequency or causality of MH in TJA. Nickel, which has the highest rate of contact sensitization among commonly used implant metals, may nevertheless be incorporated into selected alloys or occur as a trace component [48,49].

Epidemiological data underscore the clinical relevance of implant-related MH by showing that metals commonly used in arthroplasty are also among the most prevalent contact allergens in the general population. In a large multicentre European study of 3119 participants from five countries, nickel sensitization was identified in 14.5% of individuals, making it the most common metal allergen, whereas cobalt and chromium sensitization were considerably less frequent, affecting 2.1% and 0.8% of the population, respectively [50]. These findings are particularly relevant in TJA, as all three metals are constituents of commonly used orthopedic implant alloys (Table 2).

Corrosion-related release of metal ions may provoke toxic, inflammatory, or immune-mediated biological responses, but attribution of implant loosening or dermatitis to an individual metal remains difficult because corrosion products, wear particles, implant design, and host factors coexist [10,51]. Polyethylene and polymethylmethacrylate debris can provoke particle-driven foreign-body inflammation, which differs from an antigen-specific T-cell response to metal-associated antigens [48,52]. Polyethylene in particular appears unlikely to cause contact dermatitis, and current evidence supporting a true polymer allergy remains limited [52].

Despite this, hypersensitivity to bone cement has been documented. The components most often implicated in allergic reactions include benzoyl peroxide (the initiator) and N,N-dimethyl-p-toluidine (the activator) [25].

## 5. Pathophysiology

### 5.1. Cellular Mechanisms of Implant-Related Metal Hypersensitivity

All metallic biomaterials undergo some degree of corrosion when implanted in biological environments [15,53]. This process is intensified by galvanic, crevice, and fretting corrosion, as well as by mechanical wear in dynamic prosthetic components, leading to the continuous release of metal ions and particulate debris into the periprosthetic tissues and joint space [54,55]. Osteoclast precursor cells have been shown to proliferate and differentiate on the surface of metallic implants, actively contributing to degradation of prosthetic materials and further ion liberation [54,55].

MH associated with joint arthroplasty is predominantly a type IV delayed hypersensitivity reaction characterized by a cell-mediated immune response rather than an antibody-mediated mechanism [21,56,57]. Histologic evaluation of suspected implant hypersensitivity may show lymphocyte-dominated or lymphoplasmacytic inflammation, but these findings are not specific for metal allergy and must be interpreted in the context of other wear-debris reactions [56,58]. The delayed nature of the adaptive response reflects the requirement for antigen recognition and T-cell activation rather than an immediate mast-cell-mediated mechanism.

Released metal ions function as haptens, binding to endogenous proteins to form hapten–protein complexes that act as neoantigens [15,21]. These complexes are internalized and processed by antigen-presenting cells (APCs), including macrophages, dendritic cells, endothelial cells, and synovial immune cells, which subsequently present them to CD4+ T-lymphocytes [15,59]. This interaction triggers activation and clonal expansion of CD4+ and CD8+ T cells, along with macrophage recruitment and activation, resulting in the release of pro-inflammatory cytokines such as IL-1, IL-2, IL-6, TNF-α, and IFN-γ [59]. The sustained cytokine milieu promotes a chronic adaptive immune response that may damage periprosthetic soft tissues and bone [55]. Importantly, this metal-induced inflammatory response often exacerbates a pre-existing pathological state, as prior structural injuries, such as meniscal tears, initiate a feedback loop of degenerative changes and baseline immune cell activation within the joint prior to arthroplasty [60].

Persistent antigenic stimulation within the periprosthetic environment contributes to macrophage ingestion of metallic particles and subsequent osteoclast activation, ultimately promoting osteolysis and implant loosening [52,55,61,62]. Titanium and other metallic ions have been implicated in osteoclast precursor recruitment and differentiation, further accelerating periprosthetic bone resorption [55,61,62]. The intensity of the inflammatory response appears proportional to the particulate burden, with metal particles demonstrating particularly strong pro-inflammatory potential [52].

Histopathologic findings in patients with MH include perivascular lymphocytic infiltration, high endothelial venules, tissue necrosis, synovial fibrosis, granulation tissue formation, and occasional multinucleated giant cells [52,58,63]. Synovial fluid analyses frequently demonstrate elevated macrophages, polymorphonuclear leukocytes, and lymphocytes, reflecting chronic synovitis [63]. These findings support a sustained cell-mediated inflammatory process at the bone–implant interface, which may compromise vascular supply, contribute to soft-tissue damage, and accelerate implant failure [21].

Although aseptic lymphocyte-dominated vasculitis-associated lesions (ALVAL) observed in some metal-on-metal articulations share histologic similarities with delayed hypersensitivity responses, clinically relevant MH is hypothesized to involve antigen-specific T-cell responses to metal-associated antigens [21,64]. The chronicity of this immune activation in the presence of ongoing metal ion release may contribute to periprosthetic inflammation in susceptible patients, although osteolysis and implant failure are multifactorial in sensitized patients [65].

Figure 1 illustrates the proposed sequence linking implant wear and metal-ion release with antigen presentation, T-cell activation, chronic inflammation, osteolysis, and implant loosening.

### 5.2. Genetic Susceptibility and the Role of HLA in Metal Hypersensitivity

Although the cellular mechanisms of MH are increasingly well characterized, considerable interindividual variability exists in the development of hypersensitivity reactions following exposure to implant-derived metal ions. This variability suggests that host genetic factors, particularly within the HLA system, may influence susceptibility to metal-induced immune responses [31,66].

The HLA complex plays a central role in antigen presentation and T-cell activation. In delayed-type hypersensitivity reactions, peptides presented by major histocompatibility complex (MHC) class II molecules on antigen-presenting cells interact with T-cell receptors, initiating antigen-specific immune responses [67]. Metal ions can participate in this process by forming complexes with self-peptides bound within the peptide-binding groove of MHC molecules, thereby generating novel antigenic structures capable of activating T lymphocytes [31,68].

Several mechanisms have been proposed to explain how metals interact with the HLA–T cell receptor system. First, metal ions may bind directly to self-peptides presented within the MHC binding groove, forming metal–peptide complexes that function as neoantigens [68]. Second, metals may induce conformational changes in the MHC molecule itself, altering T-cell recognition [69]. Finally, metal ions may modify antigen processing pathways, resulting in presentation of otherwise cryptic self-peptides that trigger immune activation [70].

In addition to these processing-dependent pathways, a pharmacological interaction (p-i)-type mechanism should be considered. Certain metal ions, particularly nickel, can interact non-covalently and reversibly at the TCR–peptide–MHC interface and activate metal-reactive T cells without classical hapten processing. This mechanism is well described in contact-allergy models, but its quantitative contribution to periprosthetic sensitization remains uncertain [71].

Experimental studies of nickel hypersensitivity have demonstrated a strong involvement of antigen-specific CD4+ T cells in the immune response. Expansion of αβ T-cell populations reactive to nickel has been documented in both contact dermatitis and implant-associated hypersensitivity, supporting the central role of T-cell-mediated mechanisms in metal allergy [29].

At the molecular level, interactions between specific HLA class II molecules and metal-reactive T cells have been described. Structural studies have demonstrated that the MHC class II molecule HLA-DR52c (DRA*0101, DRB3*0301) can interact with T-cell receptors in the presence of nickel ions and peptide ligands, forming complexes capable of activating metal-reactive CD4+ T cells [72]. Experimental models using MHC–peptide tetramers further confirmed that nickel-specific T cells recognizing such complexes can be detected in patients with nickel sensitization and implant-related complications [29].

More recent genetic investigations have explored whether particular HLA alleles are associated with increased susceptibility to MH following joint arthroplasty. In a cohort of patients evaluated for nickel sensitization in the context of orthopedic implants, a higher frequency of the HLA-DR53 allele (encoded by DRB4*01) was observed among sensitized individuals compared with non-sensitized controls, suggesting a potential association between this allele and nickel hypersensitivity [30]. In contrast, DRB4 null variants have been described genetically, but their functional or protective significance in implant-associated metal sensitization remains uncertain [73,74].

A genetic association study involving more than 600 patients undergoing metal-on-metal hip arthroplasty further suggested that susceptibility to lymphocyte-dominated adverse reactions may reflect both metal exposure and HLA class II genotype. In particular, haplotypes containing HLA-DQA1*02:01, HLA-DQB1*02:02, and HLA-DRB1*07:01 were more frequently observed in patients who developed lymphocyte-dominated adverse reactions to metal debris [31].

These genetic observations should be interpreted as hypothesis-generating rather than as validated clinical risk markers. The DR53 report was based on a small selected subgroup (16 nickel-sensitized and 13 non-sensitized subjects) from an orthopedic referral cohort; the overall DRB4*01 frequency difference did not reach conventional statistical significance, and the authors appropriately described the finding as a potential association [30]. The Langton et al. [31] investigation was substantially larger (606 genotyped patients, including 176 with prosthetic failure and 430 asymptomatic patients) and incorporated recruitment from multiple centers, but the signal remains tied to a specific metal-on-metal hip population and has not yet been independently reproduced as a generalizable predictor across other arthroplasty cohorts. HLA linkage disequilibrium, ancestry/population structure, phenotype definitions, model-selection or multiple-testing effects, and autoimmune comorbidity therefore remain important sources of potential confounding.

One mechanistic hypothesis is that these genetic associations may be partly explained by differences in peptide-binding affinity within the HLA molecule [31]. Certain HLA class II haplotypes appear to encode peptide-binding grooves with higher affinity for metal-binding sequences of endogenous proteins, such as the N-terminal region of albumin, which contains recognized metal-binding sites [75]. If confirmed, enhanced binding of such peptides could facilitate the formation of stable metal–peptide–HLA complexes that are more efficiently recognized by T cells, thereby promoting hypersensitivity reactions [31].

Importantly, the albumin N-terminal metal-binding model should be regarded as a mechanistic hypothesis and theoretical extrapolation rather than an established causal pathway in arthroplasty. It has not been prospectively demonstrated that albumin-derived metal-binding peptides are the dominant antigens presented by the implicated HLA haplotypes in patients with implant-related MH [31].

The same HLA markers also occur in broader autoimmune and immunogenetic contexts, complicating causal attribution to metal exposure. DRB4*01/DR53 is carried in linkage disequilibrium on DRB1*04, *07, and *09 haplotypes; DR53-containing haplotypes have been associated with antiphospholipid-antibody responses, while large fine-mapping analyses in rheumatoid arthritis and systemic lupus erythematosus found that DRB1 variation accounted for MHC associations better than DRB3/4/5 and identified no independent DRB4 effect after conditioning on DRB1 [76,77]. Likewise, the DRB1*07:01–DQA1*02:01–DQB1*02:02 (DR7–DQ2.2) background has associations outside arthroplasty, including ileal Crohn’s disease, celiac susceptibility, and psoriasis [78,79,80]. DQ2.2 alone accounted for 3.4% of celiac patients in one case–control cohort and conferred relatively low risk, whereas risk increased in DQ2.5/DQ2.2 or related trans configurations [79]. These pleiotropic associations do not invalidate the arthroplasty observations, but they emphasize that a detected HLA signal may reflect linkage or a broader antigen-presentation phenotype rather than a metal-specific causal determinant.

Collectively, these findings suggest that implant-related MH results from a complex interaction between environmental exposure to metallic wear debris and host genetic susceptibility. Variability in HLA genotype may influence antigen presentation, T-cell activation, and the magnitude of the resulting immune response to metal ions released from prosthetic materials [29,30,31]. These genetic observations justify further study of immunogenetic risk stratification, but the current evidence is insufficient to support routine HLA-based implant selection [17,81].

Accordingly, HLA should be viewed as one modifier of antigen presentation, not as a necessary or sufficient determinant of sensitization. Metal responses remain strongly exposure- and context-dependent, reflecting dose, route, wear/corrosion burden, innate inflammatory signals, co-stimulation, T-cell receptor repertoire, regulatory T-cell function, age, medications, and other host factors [22,71]. Patients lacking DR53 or DR7–DQ2.2 can still develop metal-reactive T-cell responses through other class II alleles, MHC class I/CD8 pathways, and other antigen-presentation contexts. Conversely, carriage of a putative risk haplotype does not establish disease. Therefore, absence of these HLA markers must not be used to exclude MH in a symptomatic patient, and current data do not justify HLA-based reassurance or implant selection.

## 6. Clinical Presentation of Metal Hypersensitivity

The clinical presentation of MH following TJA is heterogeneous and frequently nonspecific, which contributes to the ongoing diagnostic uncertainty surrounding this condition (Table 3). In a recent referred cohort, pain was the dominant complaint, whereas eczema, instability, redness, and swelling occurred less consistently and did not reliably distinguish patients with positive patch-test findings [34]. Symptoms may develop during the first months or years after implantation [25,34]. The predominance of women in reported cohorts may partly reflect the higher background prevalence of contact sensitization, particularly to nickel, among females [50,82].

### 6.1. Articular and Periprosthetic Manifestations

Articular symptoms represent the most common clinical presentation reported in patients referred for suspected MH after arthroplasty. Patients may report persistent joint pain and swelling, accompanied by stiffness, limited range of motion, recurrent effusions, or instability [25,34,83]. These findings are nonspecific and may reflect infection, mechanical failure, adverse reactions to wear debris, or other causes of prosthetic dysfunction rather than hypersensitivity itself [25,34,83]. Generalized arthralgias have also been described, but remain uncommon and diagnostically nonspecific [25,34,84].

Clinical expression may differ according to implant location. Legacy reports describe cutaneous reactions after knee arthroplasty and deep joint pain, groin discomfort, or activity-related symptoms after hip arthroplasty, particularly with metal-on-metal bearings [64,85]. In both joint types, persistent swelling, effusion, or unexplained pain should prompt evaluation for infection, mechanical loosening, instability, adverse reactions to wear debris, and other more common causes before MH is considered.

Radiographic findings may include progressive periprosthetic lucency, osteolysis, and eventual aseptic loosening resulting from sustained inflammatory responses to metal wear products [55,63,64]. During the first several years following arthroplasty, affected patients may additionally develop chronic effusions, joint instability, and recurrent dislocation, representing further manifestations of ongoing periprosthetic tissue injury [64,86,87]. Progressive inflammation may ultimately culminate in implant loosening and failure [64,88].

### 6.2. Dermatologic Manifestations

Cutaneous manifestations constitute an important clinical clue suggestive of implant-related hypersensitivity. Dermatologic symptoms may be localized to the surgical region or become generalized, occurring in up to 35% of affected individuals [25]. Reported skin findings include erythema, papules, vesicles, induration, localized warmth, and pruritic or scaly eczematous eruptions. Less frequently, urticarial, bullous, or vasculitic lesions have been described [64,86,87].

When dermatitis is present, it may develop adjacent to the surgical incision, particularly after TKA, and can subsequently spread to distant body regions [25,34,89]. Because postoperative dermatitis has multiple possible causes, its location and timing alone do not establish implant-related hypersensitivity. Rare systemic presentations, including generalized dermatitis with alopecia, have also been described [90].

### 6.3. Adverse Local Tissue Reactions: ALVAL and Pseudotumors

Adverse local tissue reactions may become apparent during revision surgery. One lymphocyte-dominated histopathological pattern is ALVAL, which is characterized by perivascular lymphocytic infiltration and is encountered particularly in metal-on-metal articulations [64]. Although ALVAL may be compatible with a delayed-type hypersensitivity response, it is not pathognomonic and can coexist with cytotoxic and particle-driven reactions to implant debris [21,64].

Another uncommon but clinically significant manifestation is the development of pseudotumors, defined as noninfectious and nonmalignant soft-tissue masses associated with implant wear particles [91,92,93]. Patients with pseudotumors may present with hip discomfort, palpable masses, spontaneous dislocation, nerve palsy, or concomitant dermatitis [92]. Imaging studies may reveal subtle soft-tissue abnormalities, whereas the underlying pathogenesis is believed to involve both the cytotoxic effects of metal particles and delayed hypersensitivity reactions within periprosthetic tissues [94].

However, the absence of significant differences in lymphocyte transformation testing between patients with and without pseudotumors suggests that systemic delayed hypersensitivity may not represent the predominant pathogenic mechanism in all cases [95]. These observations further support the notion that metal sensitization does not invariably translate into clinically significant symptoms or implant failure [96].

Importantly, ALVAL and pseudotumors may be detected during imaging or revision work-up, but neither finding is a specific diagnostic indicator of implant allergy [21,91]. Consequently, MH remains a diagnosis of exclusion and requires careful differentiation from periprosthetic joint infection, mechanical loosening, instability, adverse reactions to wear debris, and other causes of prosthetic joint failure [7,17].

## 7. Diagnostic Methods

### 7.1. Diagnostic Approach and General Considerations

MH following total joint arthroplasty is widely regarded as a diagnosis of exclusion [7,58,97,98] and should be considered only after evaluating more common causes of implant failure, including infection, aseptic loosening, instability, and mechanical complications. Laboratory inflammatory markers, including C-reactive protein (CRP) and erythrocyte sedimentation rate (ESR), together with appropriately indicated joint aspiration, form part of the differential diagnostic work-up rather than a test for MH itself [7,98].

Currently, no single gold-standard diagnostic test exists for detecting implant-related MH [99,100]. Both in vivo and in vitro diagnostic tools have been proposed, each with important limitations regarding sensitivity, specificity, availability, and predictive value for clinical outcomes after arthroplasty [21,25].

Pre-implantation allergy evaluation should be selective and clinically contextualized rather than triggered by any nonspecific history of metal intolerance [7,21,98]. Testing may be considered when the history raises a meaningful suspicion of metal allergy; post-implantation testing may be considered in symptomatic individuals with unexplained pain, dermatitis, or implant failure only after more common causes have been evaluated [7,21,98].

### 7.2. In Vivo Testing: Skin Patch Testing

Skin patch testing remains the most commonly used in vivo method for evaluating delayed-type contact sensitization [7,86]. However, allergy-test results do not consistently correlate with peri-implant histopathology obtained during revision TKA [101]. Standardized metal preparations are applied under occlusion and reactions are graded morphologically according to erythema, infiltration, papules, and vesicles (Figure 2) [102]. The current ESCD technical recommendations on occlusion and reading times are summarized below.

According to the 2026 European Society of Contact Dermatitis (ESCD) guideline, a standard patch test uses an occlusion time of 2 days. At least two readings are recommended; ideally, reactions are assessed at Day 2, Day 3 or 4, and around Day 7. When a Day-2 reading is performed, 15–60 min should be allowed after patch removal for pressure effects to resolve. A Day-3 or Day-4 reading is essential, while the late reading improves detection of delayed reactions [103].

Importantly, a morphologically positive patch test demonstrates contact sensitization, not causality of an implant-related complication. After the final reading, the 2026 ESCD guideline recommends assessment of whether the reaction has current, past, or unknown clinical relevance. This distinction is particularly important in implant-related problems, where sensitization may coexist with mechanical failure, infection, wear-debris inflammation, or other explanations [103].

Reported estimates of the diagnostic performance of patch testing vary substantially because studies use heterogeneous reference standards and no validated clinical gold standard exists. Concordance among patch testing, lymphocyte-based assays, peri-implant histopathology, and postoperative outcomes is generally poor [17,98,101]. Accordingly, an isolated positive patch-test result should not be interpreted as diagnostic of implant-related MH.

Despite its widespread use, several limitations must be considered. The immune response elicited in patch testing is mediated primarily by cutaneous antigen-presenting cells, whereas peri-implant reactions involve macrophages and lymphocytes within the joint space, potentially reducing predictive value for prosthesis-related reactions [15]. Results are also influenced by test materials, concentrations, reading, and interpretation [63]. Although active sensitization from patch testing is possible, the 2026 ESCD guideline emphasizes that it is a very rare complication when standardized allergens and recommended test doses are used [103].

Mechanistically, the discrepancy is more substantial than a change in anatomic site: epicutaneous testing is initiated within a skin-specific antigen-presenting-cell network dominated by Langerhans cells and dermal dendritic cells, with prominent MHC class II presentation to CD4+ T cells, whereas periprosthetic tissues contain macrophages, dendritic cells, synovial/endothelial cells, and infiltrating lymphocytes in a metal-ion-rich, cytokine-dense milieu; CD8+ responses and MHC class I presentation or cross-presentation may also contribute [15,22,71]. Consequently, a positive patch test establishes cutaneous sensitization but does not reproduce the entire periprosthetic immune environment and cannot by itself prove that the implant is the causal driver of symptoms.

Pre-packaged patch-test systems do not fully cover the current European baseline series, and allergen doses may differ from investigator-loaded preparations; these limitations should be considered when interpreting negative results [103]. In some contact-dermatitis settings, standardized commercial kits such as the T.R.U.E. Test have shown limited allergen coverage and may yield false-negative reactions [104,105]. Patch testing may also be less sensitive for certain metals such as titanium because of limitations of available preparations and poor skin penetration of some salts [106,107].

The European baseline series is dynamic and requires periodic revision as exposures and evidence change [103,108]. Importantly, the 2026 ESCD guideline states that the baseline series alone is insufficient in many patients; additional allergens or special series should be selected according to the individual history and relevant exposures [103]. For suspected implant-related hypersensitivity, this supports tailoring testing to actual implant constituents and, where relevant, bone-cement components rather than relying on a fixed universal panel. The illustrative extended metal-allergen panel presented in Table 4 represents a practical testing approach currently used at our center; however, it should not be interpreted as a universally validated or ESCD-endorsed implant-allergy series.

Patch testing should be considered when delayed-type sensitization to implant materials is clinically suspected [103]. This should not be equated with universal preoperative screening or with automatic selection of a hypoallergenic implant after any positive patch test; test results must be interpreted together with the patient history, implant composition, and clinical context [21,109].

### 7.3. In Vitro Testing: Lymphocyte-Based Assays

In vitro diagnostic methods attempt to model systemic cell-mediated reactivity and include the LTT, modified lymphocyte stimulation testing (mLST), the Memory Lymphocyte Immunostimulation Assay (MELISA), lymphocyte activation testing (LAT), and separate cytokine-release or ELISA-based readouts [15,84,106].

The LTT evaluates lymphocyte proliferation after exposure to specific metal allergens, commonly quantified through thymidine incorporation [86,110]. This method offers several advantages over patch testing, including higher reported sensitivity (55–95%), quantitative output, and absence of sensitization risk due to its in vitro nature [111,112].

Because LTT is performed ex vivo, it does not expose the patient to a sensitizing challenge and generates a numerical stimulation index rather than a morphology-dependent skin reading. It is therefore an objective quantitative measure of adaptive lymphocyte reactivity to a defined metal challenge; however, a positive in vitro response demonstrates sensitization/reactivity and does not by itself establish that an implanted device is clinically causal. Conversely, diagnostic patch testing is generally safe but can very rarely induce active sensitization to a standardized allergen, a recognized in vivo complication [103].

However, reported LTT performance estimates vary with the metal tested, assay protocol, cell handling, positivity threshold, and comparator, while practical limitations include restricted allergen panels, limited availability, and the need for specialized laboratories [86,113]. Although some studies suggest that LTT may be comparable or superior to patch testing for implant-related hypersensitivity, evidence remains inconsistent and its predictive value for postoperative outcomes is unclear [15,114,115].

Modified assays such as mLST have demonstrated potential clinical relevance, with chromium sensitivity correlating with postoperative eczema following total knee arthroplasty, although supporting data remain limited [84]. The LAT has also been proposed as a monitoring tool, detecting activation markers (e.g., CD69 expression) on lymphocytes in patients with implant loosening [65].

MELISA is a modified LTT protocol designed to enhance detection of metal-reactive memory lymphocytes by using a high lymphocyte density, selected non-cytotoxic/non-mitogenic metal concentrations, morphological confirmation, and partial monocyte depletion before and after culture [106]. This monocyte-depletion step distinguishes the protocol from conventional LTT, but it also means that the assay does not reproduce the full antigen-presenting-cell-rich periprosthetic environment; moreover, evidence that MELISA is clinically superior to conventional LTT for arthroplasty-related MH remains insufficient. mLST is likewise a modification of lymphocyte stimulation conditions rather than a distinct immune mechanism, and equivalence or superiority across the available lymphocyte-based assay formats has not been established.

Published LTT/MELISA protocols and reproducibility studies have existed for many years, and quantitative sensitivity/specificity estimates have been reported in selected settings [106,111,113]. Nevertheless, the 2024 European expert consensus identified substantial inter-laboratory variation and incomplete harmonization of protocols, reference standards, and clinically validated thresholds. Accordingly, the limitation is not the absence of any standardized method, but insufficient cross-laboratory harmonization for routine arthroplasty decision-making; the expert panel therefore did not recommend LTT as a routine diagnostic allergy test for arthroplasty patients [98].

### 7.4. Leukocyte Migration and Additional Laboratory Methods

The leukocyte migration inhibition test evaluates leukocyte mobility in the presence of sensitizing antigens; decreased migration suggests hypersensitivity [15]. However, this method demonstrates limited sensitivity and is generally not recommended as a standalone diagnostic test for delayed-type hypersensitivity [15].

Available studies demonstrate poor concordance among patch testing, lymphocyte-based assays, and peri-implant histopathology [17,98,101,110]. These modalities may provide complementary information in selected patients, but none has sufficient standalone validity to confirm implant-related MH or direct revision surgery. Given the absence of a universally accepted gold-standard test, the key diagnostic considerations are summarized in Table 5.

## 8. Preoperative Metal Hypersensitivity Assessment

Current evidence does not support routine preoperative screening for MH before TJA because test results have limited and inconsistent relationships with clinically important outcomes [7,17,98,101]. Positive preoperative patch-test findings do not reliably predict implant failure or revision [17,101]. Screening questionnaires and self-reported metal intolerance should likewise be interpreted within the broader clinical context rather than used as standalone indications for hypoallergenic components [17,98].

Professional recommendations generally discourage routine preoperative testing in patients without a history suggestive of implant-material allergy, as no consistent association has been established between preoperative sensitization and postoperative cutaneous or implant-related complications [52,116].

A 2024 European expert consensus strongly rejected routine preoperative allergy screening in patients without a history suggestive of implant-material allergy. No consensus was reached for routine screening based on any history of possible allergy, whereas the panel reached 83% agreement that preimplant allergy evaluation is appropriate when the history suggests a potentially severe allergy to implant components. The experts also advised against patch testing with raw material probes or metal discs and against subcutaneous implantation of arthroplasty components as a preoperative “compatibility test” [98].

This distinction aligns with the 2026 ESCD update: patch testing is recommended when delayed-type sensitization to implant materials is clinically suspected, rather than as universal screening for all arthroplasty candidates [103].

Consequently, available evidence favors a selective, clinically contextualized testing strategy rather than universal screening or automatic testing based on any self-reported metal intolerance [7,98,101,116].

Patients who may merit more detailed preimplant evaluation include those with:

I. A documented severe or reproducible delayed reaction to metals encountered in daily life (e.g., jewelry, watches, or belt buckles) or through occupational exposure;
II. A prior patch-test-confirmed allergy to a metal or bone-cement constituent expected in the planned implant;
III. A previous suspected implant-related hypersensitivity reaction after infection and mechanical causes were excluded;
IV. A history suggesting a potentially severe allergy to a planned implant component.

In such patients, evaluation should remain targeted rather than universal and may include allergy/dermatology consultation, component-specific patch testing, and, in selected circumstances, complementary in vitro testing [7,21,98,103].

When patch testing is clinically indicated, test selection should be exposure-driven: the current European baseline series should be supplemented with relevant implant and bone-cement constituents based on the individual history and available material-composition data [103].

When preoperative testing is undertaken, evaluation should reflect the clinically relevant implant constituents and, where appropriate, bone-cement components [7,98]. Skin patch testing remains the most accessible modality; however, available studies consistently demonstrate limited ability to predict postoperative implant-related symptoms or survival [7,17,101,116]. Patients with self-reported nickel allergy generally achieve similar short-term clinical improvement and revision-free survival to patients without such a history, and current evidence remains insufficient to support routine use of hypoallergenic implants solely on this basis [81,117,118,119].

Nevertheless, hypersensitivity testing may provide clinical value in selected revision cases or in otherwise unexplained implant failure, particularly when the history, examination, implant composition, and additional investigations collectively support a clinically relevant sensitization [21,34,98]. Overall, test findings should be interpreted cautiously and integrated with clinical history, implant characteristics, histopathology where available, and surgical judgment. A diagnostic algorithm integrating current recommendations from the expert consensus on suspected metal implant allergy and the 2026 ESCD guideline for diagnostic patch testing is presented in Figure 3 [98,103].

## 9. Treatment of Metal Hypersensitivity Following Joint Arthroplasty

### 9.1. General Principles and Conservative Management

Management of suspected MH after joint arthroplasty remains challenging. MH should be considered a diagnosis of exclusion after infection, mechanical failure, adverse reactions to wear debris, and other common causes of implant-related symptoms have been evaluated [7,98,99]. Because treatment evidence is limited and heterogeneous, therapeutic decision-making must be individualized [7,98].

First-line management is typically conservative and symptom-directed. Nonsteroidal anti-inflammatory drugs, physical therapy, and appropriately selected intra-articular corticosteroid injections may provide relief in patients presenting with joint pain and swelling. When cutaneous manifestations occur, treatment strategies used for allergic contact dermatitis, including topical corticosteroids and topical calcineurin inhibitors, may be considered; systemic treatment should be reserved for appropriate clinical indications [120]. Referral to dermatology or allergy specialists is advisable for persistent localized or systemic dermatitis and when formal testing is being considered [7,98].

For rare severe generalized or systemic manifestations, short courses of systemic corticosteroids or other syndrome-directed immunomodulatory therapy may be required under dermatology/allergy supervision; antihistamines may be used for symptomatic pruritus or urticarial features but do not treat the underlying type IV T-cell response. Evidence specific to implant-associated MH is largely case-based, and systemic therapy should not delay evaluation for infection, mechanical failure, or removal/revision of a clearly implicated device [121].

Short courses of topical corticosteroids have demonstrated clinical improvement in some patients with localized dermatitis, highlighting the potential effectiveness of non-surgical management in selected cases [89,122].

### 9.2. Indications for Surgical Intervention and Revision Arthroplasty

Surgical intervention should be considered cautiously and generally reserved for patients with persistent symptoms accompanied by objective implant-related pathology after failure of conservative treatment and exclusion of more common causes [7,52,85,89,98]. Revision based solely on unexplained pain or an isolated positive allergy test is unlikely to provide predictable benefit and is therefore discouraged [52,98].

When hypersensitivity-mediated implant failure is strongly suspected, revision to components lacking the offending allergen has been associated with symptom resolution in multiple reports [63,89,123]. Case studies have demonstrated rapid improvement of dermatitis following removal or replacement of sensitizing implants, supporting a causal relationship in selected patients [121,122]. However, revision outcomes may be inferior compared with revisions performed for other indications, potentially due to preexisting soft tissue damage and osteolysis [91,124].

Early recognition of hypersensitivity-related implant failure has therefore been emphasized to limit tissue destruction and improve revision outcomes [124].

### 9.3. Hypoallergenic Implants and Material Considerations

The use of hypoallergenic implant components remains controversial due to limited long-term outcome data and challenges in accurately diagnosing metal allergy. So-called hypoallergenic options include alternative-material components or standard cobalt–chromium components with surface coatings intended to reduce exposure to sensitizing metals [125,126,127].

Mechanistically, titanium-nitride/titanium-niobium-nitride coatings, oxidized-zirconium or zirconium-based surface technologies, and experimental diamond-like-carbon coatings can function as wear/corrosion barriers that reduce release of cobalt, chromium, or nickel ions from the substrate and may thereby reduce formation of metal–protein hapten complexes. The protective effect depends on coating integrity, and wear, defects, or delamination can diminish the barrier function [128].

Examples of alternative components include ceramic-on-polyethylene or ceramic-on-ceramic bearings in THA and selected titanium-alloy, oxidized-zirconium, or coated components in TKA [7,26,119]. Randomized evidence comparing coated and standard TKA implants has demonstrated minimal clinically relevant differences in outcomes and systemic metal-ion levels, although isolated increases in chromium have been reported without clear clinical significance [20,126].

Importantly, trace metal exposure may persist despite revision procedures, as nickel-containing surgical instruments and small quantities of nickel present in titanium alloys may contribute to continued exposure; the clinical relevance of these findings remains uncertain [25].

### 9.4. Alternative and Emerging Treatment Strategies

In selected patients, removal of temporary hardware or revision to components that avoid a clinically relevant sensitizing metal may be considered when a causal relationship is strongly supported. Titanium-based components are often considered because of their relatively low allergenic potential, although hypersensitivity-like reactions have been reported after Ti-6Al-4V osteosynthesis and in other implant settings; no implant material should therefore be described as completely non-allergenic [129,130]. Such case-based evidence supports biological plausibility but does not establish the incidence of titanium hypersensitivity after TJA. Coated implants and alternative bearing surfaces continue to be investigated as strategies to reduce metal exposure [131].

Experimental approaches such as oral desensitization have shown preliminary benefit in reducing dermatitis symptoms in nickel-allergic individuals, although relapse following treatment discontinuation has been reported, and routine use cannot currently be recommended [132].

Because TNF-α, IL-1, and IL-6 participate in periprosthetic inflammatory networks, targeted biologic blockade is mechanistically conceivable. However, there is currently no validated clinical evidence supporting anti-TNF, anti-IL-1, or anti-IL-6 therapy specifically for implant-related MH; such treatment should therefore be regarded as experimental/research-based rather than an alternative to correcting an implicated implant or another established cause of failure [15,55].

### 9.5. Clinical Considerations and Patient Counseling

Given the limited and heterogeneous evidence base, patients should be counseled regarding the uncertain benefits and unpredictable outcomes of revision surgery for suspected MH [7,23,98]. Surgical treatment should be undertaken only when a coherent association between hypersensitivity and objective implant-related pathology is established, and revision should address all implicated components with appropriate alternative materials where feasible [7,98,125].

Overall, current evidence supports a stepwise treatment strategy, beginning with conservative management, progressing to dermatologic therapy for cutaneous disease, and reserving revision arthroplasty with hypoallergenic implants for carefully selected patients with persistent symptoms or implant failure attributed to hypersensitivity. A treatment algorithm integrating current recommendations from the expert consensus on suspected metal implant allergy and the 2026 ESCD guideline for diagnostic patch testing is presented in Figure 4 [98,103].

## 10. Future Directions

Despite progress in understanding implant-related MH, major knowledge gaps remain. The foremost limitation is the absence of a validated diagnostic reference standard. Current modalities, including in vivo patch testing and in vitro lymphocyte-based assays, show variable performance and limited clinical predictive value; for lymphocyte-based assays, the more precise concern is incomplete inter-laboratory harmonization and the lack of universally validated clinical decision thresholds rather than the absence of published assay protocols. Developing prospectively validated diagnostic pathways that integrate clinical phenotype, implant exposure, histopathology, and immune testing should therefore be a research priority.

The EAACI 2023 nomenclature offers a useful framework for hypothesis generation, but implant-related MH should not be assumed to map uniformly onto all type IV subcategories. Classical metal contact allergy is most closely aligned with a T1-dominant response, whereas the contribution of T2- or T3-dominant pathways to periprosthetic disease remains insufficiently defined. Future studies should test these mechanisms directly using standardized tissue and blood phenotyping rather than extrapolating them from other allergic diseases.

Host genetic susceptibility, including HLA class II variation, is a promising but still preliminary research direction. Large prospective multicenter studies should combine HLA typing with quantitative metal exposure, standardized clinical phenotyping, histopathology, T-cell receptor profiling, and contemporary molecular approaches such as single-cell or spatial immune analysis. This broader strategy would help determine whether HLA associations are independent predictors, markers of specific immune endotypes, or correlates of other exposure-related factors. Future HLA studies should pre-specify ancestry and population-stratification methods, report and adjust for relevant autoimmune comorbidities, account for linkage disequilibrium and multiple testing, quantify metal exposure, and require independent external replication before any genotype is used for clinical risk stratification [76,77,78,79,80].

If these relationships are validated, integrated immunogenetic and biomaterial profiling could eventually contribute to precision risk stratification. At present, however, the evidence does not support routine HLA-based selection of arthroplasty components.

## 11. Conclusions

MH following TJA is an uncommon and clinically challenging diagnosis. Antigen-specific, delayed T-cell responses to metal-associated antigens may contribute in susceptible patients, but they coexist with nonspecific inflammatory responses to wear and corrosion products. Persistent pain, dermatitis, synovitis, osteolysis, and loosening are therefore not specific for MH and require careful differential diagnosis.

In contemporary practice, suspected MH remains a diagnosis of exclusion. Infection, instability, aseptic loosening, and other mechanical or inflammatory causes should be evaluated before symptoms are attributed to metal allergy. Routine preoperative allergy screening in patients without a suggestive history is not recommended; even in patients reporting possible metal allergy, testing should be guided by the severity and clinical relevance of the history rather than used as a universal compatibility screen.

HLA associations should therefore be interpreted as preliminary modifiers of susceptibility rather than deterministic biomarkers; absence of a reported risk allele does not exclude MH, and carriage of such an allele does not establish implant causality.

Management should be individualized and stepwise. Conservative and dermatologic therapy may be appropriate for selected symptoms, whereas revision to alternative-material components should be reserved for carefully selected patients in whom a clinically meaningful relationship between the implant and hypersensitivity is strongly supported. Progress toward precision arthroplasty will require better phenotyping, standardized diagnostics, and prospective validation of molecular and immunogenetic biomarkers.

## Figures and Tables

**Figure 1:**
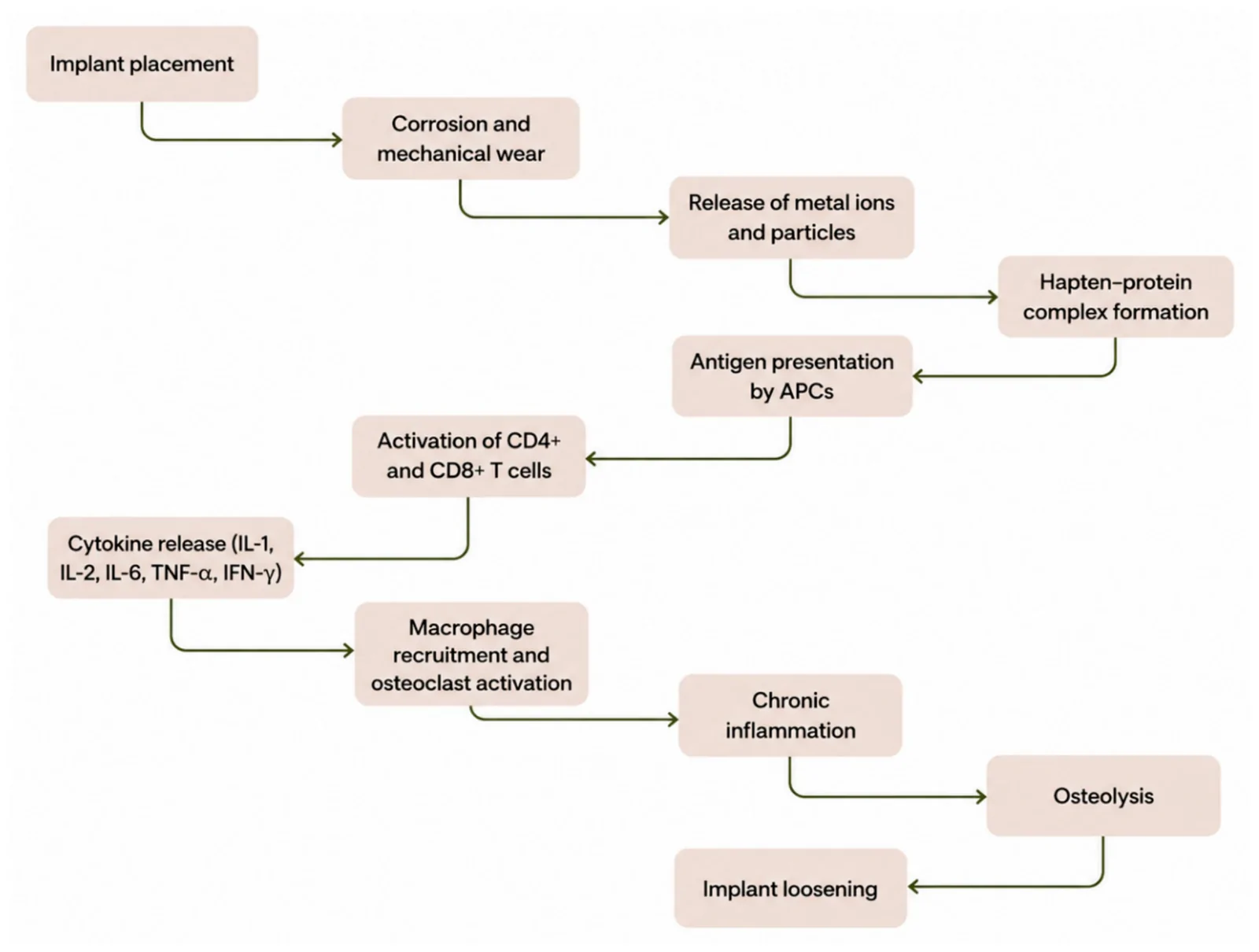
Proposed sequence of events during the immunopathogenesis of implant-related metal hypersensitivity.

**Figure 2:**
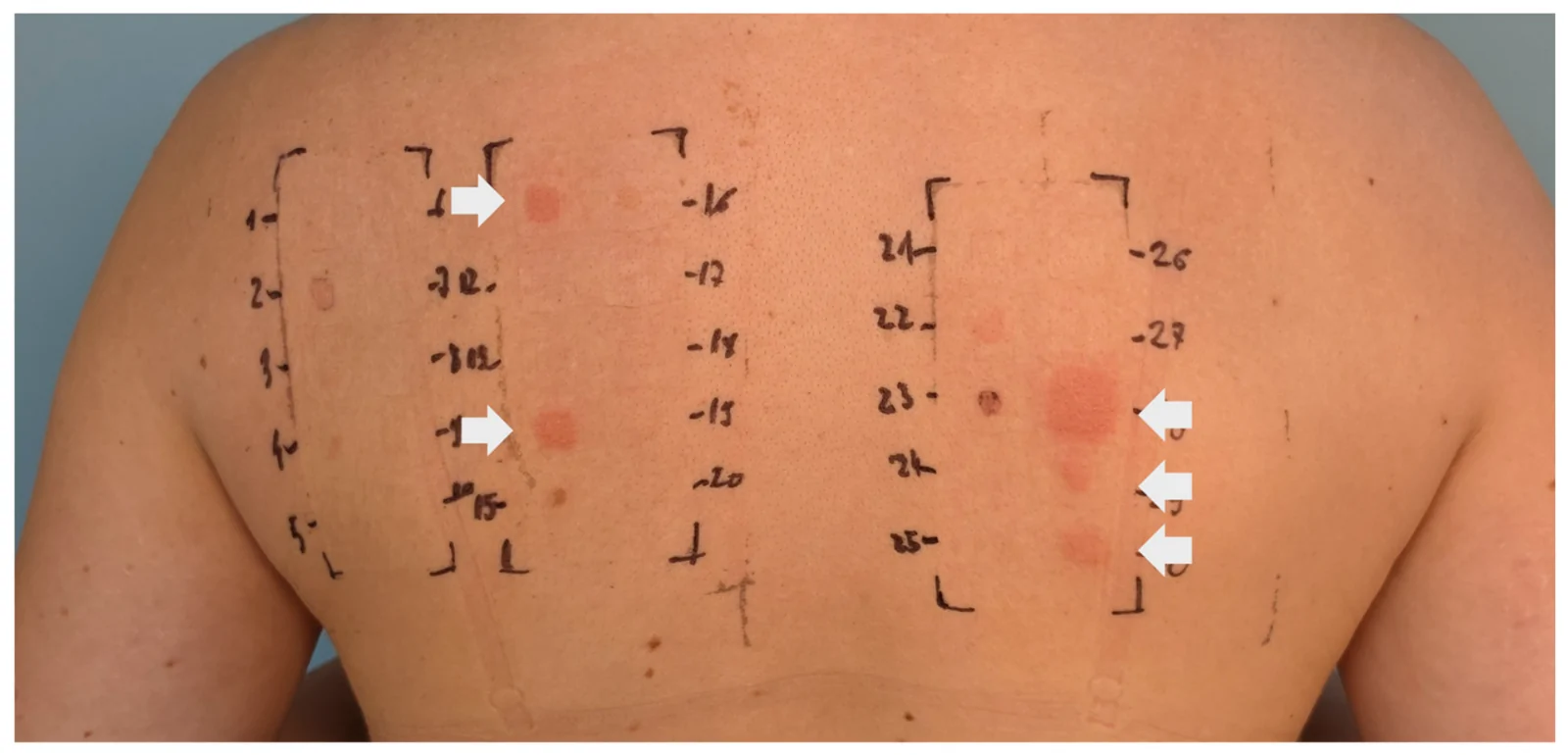
Representative patch-test image. Reading after 72 h shows positive results with 5 haptens (indicated by white arrows). Photograph taken by K.P.-K., patient consented for photography and publication.

**Figure 3:**
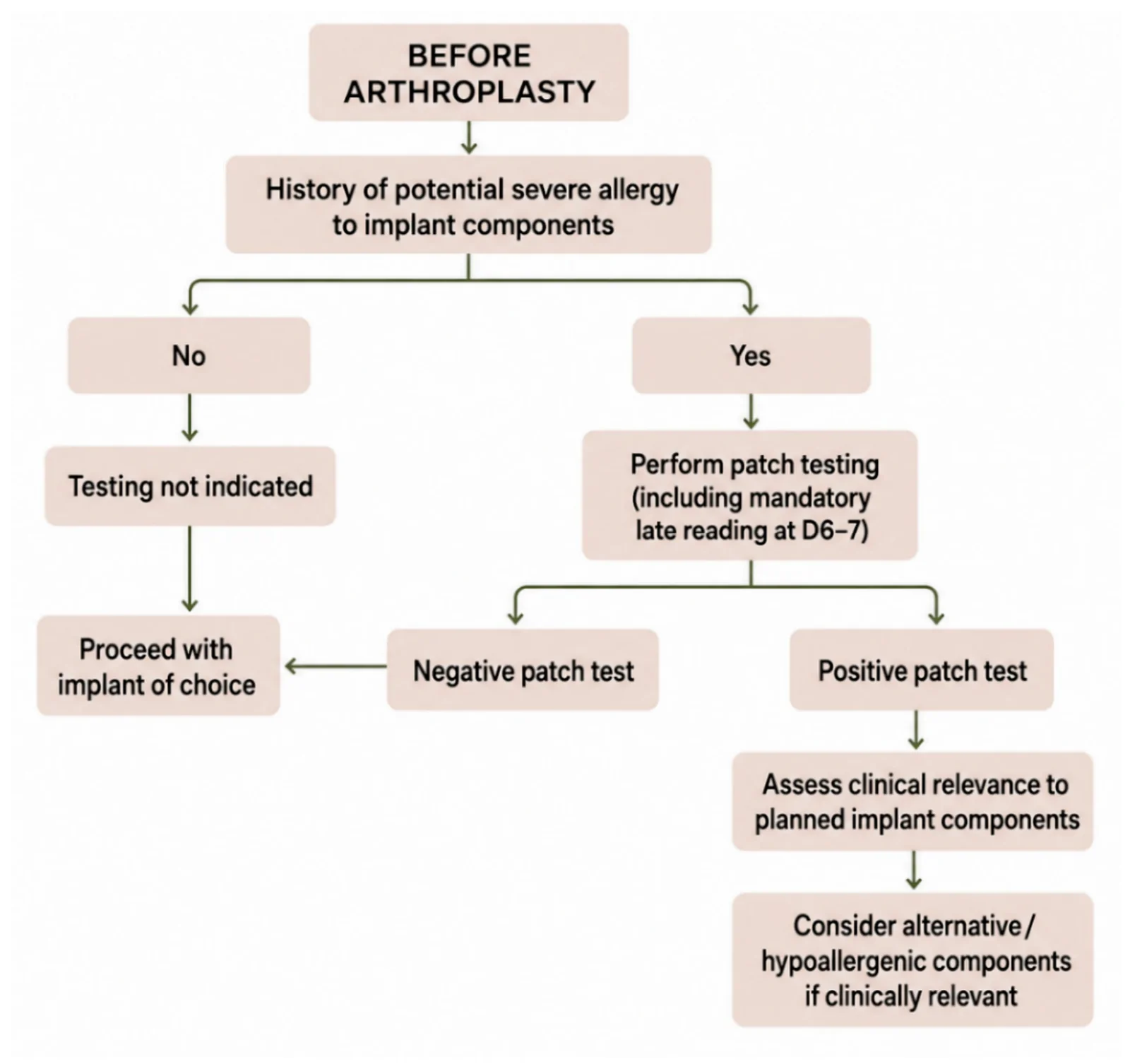
Diagnostic algorithm for metal hypersensitivity.

**Figure 4:**
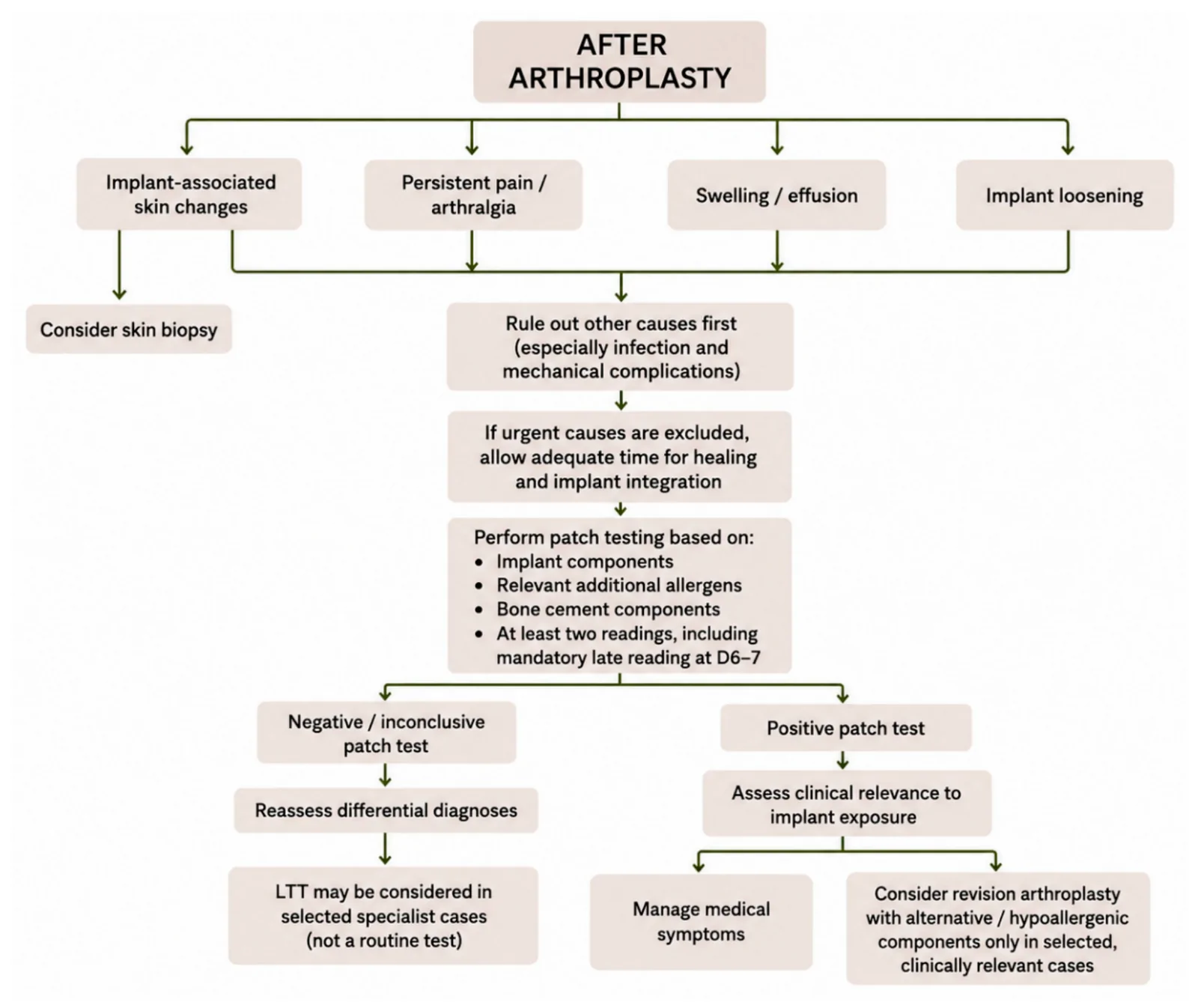
Metal hypersensitivity treatment algorithm.

**Table 1:** Immunological Features of Type IV Hypersensitivity Subtypes (IVa–IVc).

Feature	Type IVa (T1 Response)	Type IVb (T2 Response)	Type IVc (T3 Response)
Dominant immune cells	Th1, Tc1 cells, macrophages	Th2 cells, ILC2, eosinophils	Th17, Tc17, ILC3 cells, neutrophils
Key cytokines/mediators	IL-12 and IFN-γ (T1 polarization/activation); IL-23 (maintenance/effector support); TNF-α, lymphotoxin	IL-4, IL-5, IL-13	IL-17 family cytokines, IL-8
Main effector mechanisms	Macrophage activation, granuloma formation, Tc1-mediated cytotoxicity	IgE class switching, eosinophil activation and survival	Neutrophil recruitment and activation
Typical clinical manifestations	Allergic contact dermatitis, chronic hypersensitivity pneumonitis, celiac disease	Asthma, allergic rhinitis, chronic rhinosinusitis, atopic dermatitis	Neutrophilic inflammatory diseases

**Table 2:** Prevalence of Sensitization to Metals Commonly Used in Orthopedic Implants in the European General Population (EDEN Fragrance Study). Co, cobalt; Cr, chromium; Mo, molybdenum.

Metal	Typical Presence in Arthroplasty Implants	Prevalence of Sensitization (%)
Nickel	Stainless steel; some Co–Cr alloys; trace contamination may occur in other alloys	14.5
Cobalt	Co–Cr–Mo alloys	2.1
Chromium	Stainless steel; Co–Cr–Mo alloys	0.8

**Table 3:** Clinical Manifestations and Associated Findings in Suspected Metal Hypersensitivity Following Total Joint Arthroplasty.

Clinical Domain	Most Common Manifestations
Articular/periprosthetic	Pain, swelling, stiffness, effusion, synovitis, osteolysis, aseptic loosening
Dermatologic	Eczema, erythema, papules, vesicles, pruritus, generalized dermatitis
Adverse local tissue reactions (not specific for MH)	ALVAL, pseudotumors, osteolysis, instability, recurrent dislocation, implant failure

**Table 4:** Illustrative Extended Metal-Allergen Panel for Implant-Related Patch Testing.

No.	Allergen	Concentration
1.	Potassium dichromate	0.5%
2.	Cobalt(II) chloride hexahydrate	1.0%
3.	Nickel sulfate hexahydrate	5.0%
4.	Copper sulfate	2.0%
5.	Sodium tetrachloropalladate hydrate	3.0%
6.	Aluminum chloride hexahydrate	2.0%
7.	Vanadium(III) chloride	1.0%
8.	Manganese(II) chloride	2.0%
9.	Tungsten	5.0%
10.	Iron(III) chloride	2.0%
11.	Zirconium chloride	1.0%
12.	Titanium nitride	5.0%
13.	Titanium oxalate	5.0%
14.	Niobium chloride	0.2%
15.	Silver nitrate	1.0%
16.	Ammonium tetrachloroplatinate	0.1%
17.	Molybdenum	5.0%
18.	Zinc	2.5%
19.	Sodium gold thiosulfate	2.0%
20.	Iridium	1.0%

**Table 5:** Key Diagnostic Considerations in Suspected Metal Hypersensitivity After Total Joint Arthroplasty.

Diagnostic Aspect	Key Point
Diagnosis	MH remains a diagnosis of exclusion.
Initial evaluation	Exclude infection, aseptic loosening, instability, and mechanical causes first.
Gold-standard test	No universally accepted gold-standard diagnostic test exists.
Patch testing	Standard in vivo test for contact sensitization; useful when delayed-type sensitization to implant materials is clinically suspected, but positive reactions require assessment of clinical relevance and do not by themselves establish implant causality.
LTT	Objective, quantitative in vitro measure of metal-specific adaptive lymphocyte proliferation that avoids in vivo sensitization and may complement patch testing in selected cases; published protocols and performance estimates exist, but inter-laboratory harmonization, reference standards, and clinically validated outcome thresholds remain insufficient for routine stand-alone arthroplasty decision-making.
Histopathology	Findings such as ALVAL may support the diagnosis but are not pathognomonic.
Clinical interpretation	Always interpret test results in conjunction with clinical presentation, implant characteristics, and other diagnostic findings.

## Data Availability

No new data were created or analyzed in this study. Data sharing is not applicable to this article.

## Abbreviations

The following abbreviations are used in this manuscript:

ALVAL	Aseptic lymphocyte-dominated vasculitis-associated lesions
APC	Antigen-presenting cell
CD	Cluster of differentiation
Co-Cr-Mo	Cobalt–chromium–molybdenum
CRP	C-Reactive Protein
EAACI	European Academy of Allergy and Clinical Immunology
ELISA	Enzyme-Linked Immunosorbent Assay
ESCD	European Society of Contact Dermatitis
ESR	Erythrocyte Sedimentation Rate
HLA	Human leukocyte antigen
IFN-γ	Interferon-gamma
Ig	Immunoglobulin
IL	Interleukin
ILC	Innate lymphoid cell
LAT	Lymphocyte activation test
LTT	Lymphocyte transformation test
MELISA	Memory Lymphocyte Immunostimulation Assay
MH	Metal hypersensitivity
MHC	Major histocompatibility complex
mLST	Modified lymphocyte stimulation testing
Tc	Cytotoxic T cell
Th	T helper cell
TJA	Total joint arthroplasty
TNF-α	Tumor necrosis factor-alpha
